# Competition or Complementarity Among Telemedicine Tools in Ambulatory Care Practice: Cross-Sectional Analysis

**DOI:** 10.2196/75246

**Published:** 2025-12-23

**Authors:** Xiang Oliver Liu, Avijit Sengupta

**Affiliations:** 1 Business Information Systems, Business School The University of Queensland Brisbane, Queensland Australia; 2 Centre for the Business and Economics of Health, Business School The University of Queensland Brisbane, Queensland Australia

**Keywords:** office-based physicians, ordered logit regression model, ordered probit regression model, percent of patient visit, physicians’ satisfaction, quality of care, telemedicine technology tools

## Abstract

**Background:**

Telemedicine use surged due to its capacity to deliver safe, remote care. As the public health crisis subsides, evaluating the interplay among various tools, such as video, audio, and text, becomes critical to sustained use. With health care shifting back to in-person models, understanding whether telemedicine tools complement or compete provides valuable insights for future technology design and usage strategies.

**Objective:**

This study investigates whether different types of telemedicine technology tools complement or compete while physicians deliver health care services through them. A clear understanding of the relationships between telemedicine technology tools, physicians’ satisfaction, evaluation of care quality, and patient visit percentages is crucial for the design of new telemedicine technology platforms and ensuring quality of care services through technology platforms.

**Methods:**

To fulfill our objective, we analyzed data from the 2021 National Electronic Health Records Survey. We used ordered logit and probit regression models to evaluate the effects of telemedicine technology tools on physicians’ overall satisfaction, quality of health care evaluation, and the percentage of patient visits via telemedicine.

**Results:**

A total of 1875 office-based physicians in the United States completed the survey. Three main outcomes were assessed, including physician satisfaction (n=1614), evaluation of health care quality (n=1617), and the percentage of patient visits conducted via telemedicine (n=1558). Ordered logit and probit regression analyses revealed that the aggravated use of telemedicine tools had a significant impact on improvements in all 3 outcomes. A unit increase in telemedicine tools was associated with a 4.2 percentage point increase in the predicted probability of physicians being “very satisfied” (*P*<.001) and a 5.2 percentage point increase in evaluating telemedicine quality as “to a great extent” (*P*<.001). For patient visits, a unit increase in telemedicine tools was associated with a 1.8 percentage point increase in the likelihood of reporting “≥75% of visits via telemedicine” (*P*<.001). Disaggregated analysis indicated that all individual tools were positively associated with physician satisfaction and quality evaluation (*P*<.05). Bundle models revealed patterns consistent with complementarity (several bundles exceeded their constituent tools) and competition (some significant bundles were smaller than at least one constituent tool), aligning with the presence of both reinforcing and overlapping functionalities.

**Conclusions:**

Our study demonstrates that telemedicine tools interact in ways that can be either complementary or competitive, depending on how their functionalities align within physicians’ workflows. Videoconferencing tools, especially when integrated with electronic health record platforms, act as a central complementary component that enhances physicians’ satisfaction and evaluation of care quality. In contrast, combinations lacking video capability or involving multiple nonintegrated platforms fragment workflows and increase cognitive burden. These findings emphasize the importance of designing telemedicine tool bundles that align media capabilities with clinical communication needs, thereby improving satisfaction and supporting sustainable, high-quality telemedicine practice.

## Introduction

### Overview

Telemedicine, the use of telecommunications technology to provide health care remotely [1], has rapidly evolved to improve access to high-quality, efficient, and cost-effective health care, particularly during a major public health crisis like the COVID-19 pandemic [2,3]. Due to the unique characteristics of telemedicine, it has emerged as an ideal solution for mitigating the risks of secondary virus transmission and continuity of health care services. During the COVID-19 pandemic, there was a significant surge in demand for telemedicine, leading to substantial growth in the telemedicine industry, with remote appointments increasing by 154% within a year [4]. Meanwhile, the global telemedicine market is projected to grow from US $60.15 billion in 2023 to US $309.90 billion by 2032, with a compound annual growth rate of 19.98% [5].

Although telemedicine adoption was initially slow [6] and hindered by restrictive administrative regulations as well as limited financial investment [7], the COVID-19 pandemic significantly accelerated its adoption as it became the only alternative to in-person care, offering the safest mode of health care delivery [8]. This shift relied on using multiple telemedicine tools, which provided physicians and patients with multiple synchronous and asynchronous communication channels, including audio, video, and text (with and without electronic health records [EHRs]).

During and post the COVID-19 pandemic, most physicians received access to more than one type of telemedicine tool, which usually supports different functionalities (eg, telephone, videoconferencing, platforms without EHR, and platforms with EHR). Considering that individual tools and their combinations differ in efficacy and consequently influence the quality of health care delivery, it is essential to identify their performance both as independent tools and as integrated bundles.

However, there are only a handful of studies that have compared different telemedicine tools before or after the COVID-19 pandemic. For instance, the meta-analysis by Byambasuren et al [9] revealed no significant difference in clinical effectiveness between telephone and video consultations, though video was preferred for clinical assessment despite feasibility issues. In a separate study, Alhajri et al [10] found that physicians had greater confidence in acute care and patient education with video compared to audio but experienced longer consultation times. In addition, Orrange et al [11] suggested that most patients preferred video over telephone for telehealth visits despite encountering technical issues, due to the overall convenience. Collectively, these studies highlight that different telemedicine tools have unique strengths and limitations, which are shaped by user and usage context, as well as the mode of interaction. However, we still have very little knowledge about their perception and performance when they are deployed together.

Additionally, although studies compare different telemedicine tools from various perspectives, they often overlook the communication functions embedded within each tool. Communication is the core function of telemedicine tools, particularly crucial for ambulatory care physicians who face challenges related to technology infrastructure, such as low bandwidth and slow internet speeds [12]. While previous studies have compared different telemedicine tools, such as audio and video, in terms of perceived convenience, clinical effectiveness, or health care quality, they have mostly treated these tools as individual units.

Surprisingly, there is little exploration that investigates how these telemedicine tools, with their varying levels of media richness, synchronicity, and interactivity, work together and independently to influence clinical communication. For example, both Doxy.me (a telemedicine platform) and FaceTime are labeled as “video” tools. However, they may differ substantially in how they support real-time feedback, offering visual cues, or in interface stability. These communication functionalities, rather than the tool types themselves, shape how effectively clinical conversations are conducted and decisions are made. Therefore, it is also important to investigate whether telemedicine tools using different communication modes function in a complementary or competitive manner with existing physician workflows [13].

If these tools are complementary, they can collectively enhance health care delivery by improving system efficiency, ensuring better alignment between physicians’ tasks and telemedicine tools, and providing a seamless experience that increases physician satisfaction [14]. On the other hand, if they compete, this may result in duplicated functionalities, fragmented workflows, increased cognitive burden, and communication fatigue, ultimately reducing satisfaction and the effective use of telemedicine [15].

Thus, understanding the nature of the relationship (complementary vs competitive) between telemedicine tools helps inform sustainable telemedicine use, especially when it is no longer a necessity but an optional provision. For a complementary relationship, there are different usage strategies and policies for physicians, clinical settings, or industry, compared to a competitive relationship. This leads us to posit our research question: “Do telemedicine tools compete or complement each other?”

In our study, we aimed to answer this question from the perspective of physicians, the primary users of telemedicine tools. We used data from the 2021 National Electronic Health Records Survey (NEHRS) [16-19] and applied media synchronicity theory (MST) to answer the research question. We used ordered logit and probit regression models for empirical analysis.

Our findings reveal that while both independent and combined uses of telemedicine tools enhance physicians’ satisfaction and quality-of-health care evaluations, their combinations are not always complementary. Instead, various telemedicine bundles produce either complementary or competing effects depending on how functionalities align within physicians’ clinical workflows. Specifically, video-based communication tools serve as a central complementary element that enriches clinical assessment and communication, particularly when integrated with EHR-embedded platforms. However, bundles lacking video capability or comprising multiple nonintegrated platforms, like the simultaneous use of EHR-embedded and non–EHR-embedded telemedicine platforms, lead to workflow fragmentation and cognitive overload, ultimately lowering physicians’ satisfaction and their evaluations of care quality. In addition, tool bundles with a higher number of telemedicine tools increase the percentage of patients who visit physicians via telemedicine by enhancing flexibility and accessibility. Based on our findings, we recommend telemedicine technology vendors and platform developers for technological systems designed to ensure the complementary behavior of different tools based on physicians’ specific demands.

### Aim

Understanding whether telemedicine tools operate in complementary or competing manners is essential for sustained use of telemedicine in the postpandemic era. This study examines the impact of individual telemedicine tools and combinations of tools on physicians’ satisfaction, perceived quality of care, and the proportion of patient visits conducted via telemedicine. Specifically, we investigate whether different combinations of telemedicine tools function as complements or substitutes within the clinical workflows of ambulatory care physicians.

### Literature Review

Although telemedicine technology has existed for decades, it was not widely adopted by physicians or patients until the pandemic. In our literature review section, we primarily focus on (1) the technological capabilities of telemedicine tools and their associated tasks, (2) the impact of telemedicine technologies on physicians’ satisfaction, (3) the impact of telemedicine technologies on physicians’ evaluation of telemedicine service quality, and (4) the impact of telemedicine technologies on patients’ visits choices.

#### Telemedicine Technology Capabilities and Their Associated Tasks

Telemedicine tools can be grouped into 2 categories based on their synchronous (eg, real-time audio and video consultations) or asynchronous (eg, message-based or store-and-forward systems) communication features. Each telemedicine tool supports specific health care tasks depending on its technical features, such as media richness, interactivity, and synchronicity. Synchronous telemedicine tools, such as live video consultations or real-time chat systems, are widely accepted for their ability to facilitate immediate interaction between physicians and patients [20]. These tools aim to replicate in-person visits, enabling real-time diagnosis and immediate medical advice through audio and video communication channels [21-23]. However, they are dependent on both parties’ (physician and patient) simultaneous availability. Alternatively, asynchronous telemedicine tools, like email, offer flexibility and convenience by enabling patients and health care providers to communicate without the need for simultaneous presence [24,25]. These tools are especially effective for follow-up care, chronic disease management, or sharing test results, where real-time interaction is not necessary. Their acceptance is high among patients seeking time-efficient solutions or health care providers managing a large number of patients. However, the lack of immediate feedback limits their effectiveness for patients who require urgent attention. Therefore, it is essential to strike a balance between the needs of physicians and patients when implementing telemedicine tools.

#### Impact of Telemedicine Technologies on Physicians’ Satisfaction

Physician satisfaction is a crucial determinant of continuous telemedicine adoption and long-term system use [14]. Prior studies suggest that physicians’ satisfaction is shaped by both system-level attributes, such as usability, interoperability, and reliability, as well as user-level experiences, such as workload, communication quality, and perceived clinical control [26,27]. For example, video-based telemedicine generally contributes to higher physicians’ satisfaction levels than telephonic consultations, as it allows richer interactions, visual assessment, and enhanced rapport with patients [10]. However, video consultations are also associated with longer session times and potential technical issues, which can diminish physicians’ satisfaction under certain conditions [28,29]. Therefore, current studies indicate that video should supplement, rather than replace, the telephone [28,29].

Moreover, when multiple tools are available, physicians’ satisfaction depends on whether these telemedicine tools are seamlessly complementary and align with physicians’ health care–related task requirements [27]. In contrast, physicians might report frustration when tools compete, for example, when an EHR-integrated video platform and an independent messaging app duplicate documentation or scheduling tasks [30].

#### Impact of Telemedicine Technologies on Physicians’ Evaluation of Telemedicine Service Quality

Physicians’ evaluation of telemedicine service quality reflects their comparison of the perceived quality of health care between telemedicine and traditional in-person visits [31]. Studies have shown that asynchronous tools, such as email, are valued for follow-up communication; however, their effectiveness in immediate medical decision-making is lower than that of face-to-face methods [32]. In contrast, synchronous video consultations enhance physicians’ diagnostic confidence and clinical effectiveness. In particular, for acute care or clinical assessments, the quality of synchronous video consultations can be comparable to that of traditional in-person care, especially under acute external stress, such as during the pandemic period [28,33]. Furthermore, the integration of EHRs can improve physicians’ perceived quality of care by facilitating decision-making through access to and sharing comprehensive patient medical information, thereby ensuring continuity of care [34,35]. However, excessive system complexity or poor interoperability between platforms can have the opposite effect, increasing physicians’ cognitive load, disrupting clinical workflows, and ultimately reducing their evaluation of telemedicine service quality [36,37].

#### Impact of Telemedicine Technologies on Patients’ Visit Choices

The adoption of telemedicine technologies has also influenced patients’ preferences and behaviors regarding the types of visits they prefer. For example, the availability of video and audio consultations increases health care access by reducing travel time, waiting time, and perceived risk of exposure [33,38,39]. Therefore, during the COVID-19 pandemic, patients widely adopted telemedicine as a substitute for in-person visits, with remote consultations dramatically increased across multiple specialties [8]. Postpandemic studies, however, indicate that visit choices now depend on perceived convenience, trust, and the nature of the medical issue [40].

Patients generally prefer video consultations over audio when visual assessment or relational communication is important; however, they choose telephone consultations for brief follow-ups or low-complexity issues [41]. Significantly, patients’ continued usage in telemedicine is shaped by their communication experience, the reliability of the technology, and the perceived competence of the physician [42].

To provide an overview of the existing literature in this domain, we synthesized the relevant literature into Multimedia Appendix 1. Our review reveals that while literature often examines individual telemedicine tools or analyses multiple tools separately within a study, there is limited research investigating whether multiple tools complement or compete with each other.

This knowledge is critical, as understanding the interplay between telemedicine tools could provide insights into their collective impact on physicians’ overall satisfaction, evaluations of health care quality, and the proportion of patient visits conducted via telemedicine. To address this knowledge gap, our study explores whether complementary or competitive relationships among various telemedicine tools influence the outcomes. We aim to contribute to the telemedicine literature, benefiting health care professionals, researchers, and designers of health IT alike.

### Theoretical Framing

To better explore whether telemedicine tools compete or complement each other, we applied MST. MST emphasizes how different media capabilities support 2 fundamental communication processes: conveyance and convergence [43,44]. Conveyance involves transmitting complex information that requires the receiver to process and interpret it, whereas convergence aims to establish a shared understanding and reach a consensus among participants. Additionally, MST identifies five core media capabilities: (1) symbol sets, the variety of ways information can be encoded (eg, text, voice, video); (2) parallelism, the extent to which multiple conversations can occur simultaneously; (3) transmission velocity, how quickly messages are delivered; (4) rehearsability, the ability to fine-tune a message before sending it; and (5) reprocessability, the extent to which a message can be reviewed or revisited after transmission.

In the context of telemedicine, different tools embody distinct combinations of these capabilities and therefore serve different communicative purposes. For example, telephone audio (T1) offers low symbol diversity and reprocessability, making it suitable for simple updates rather than complex discussions. Videoconferencing tools (T2), with richer symbol sets and high transmission velocity, support synchronous interactions, visual assessment, and real-time convergence between physicians and patients. Non–EHR-integrated telemedicine platforms (T3) support structured, health-specific interactions but often lack continuity of documentation or direct access to clinical records, which limits their reprocessability and integration. However, they may offer higher parallelism and flexibility, especially for lightweight or routine consultations. EHR-integrated telemedicine platforms (T4) enhance both rehearsability and reprocessability by enabling real-time clinical documentation, access to patient history, and retrospective review. However, they also significantly increase the cognitive load on physicians [44] when navigating patient information in an EHR system, which remains a demanding task. Therefore, we argue that no single telemedicine tool is suitable for all tasks in all care contexts. Based on the above argument, telemedicine tools can be selected or combined to optimize communication according to the requirements of clinical or administrative tasks. MST thus provides a useful lens for interpreting how these tools complement or compete within ambulatory care, in the context of physicians’ workflows.

Previous research has reported the application of MST in the domain of telemedicine. Tan and Yan [45] used MST to classify text and patterns in recognition, while Yan et al [46] used MST to design a comparative case study and conduct a deductive analysis of teleconsultation adoption in clinical settings. Tan and Yan [45] demonstrated that physicians’ response behaviors and media features, particularly response speed and the availability of voice services, jointly influenced their perceived service quality in mobile health care, with media synchronicity capabilities acting as a moderating factor. Yan et al [46] suggested that when the media synchronicity of the adopted technology matches the synchronicity requirements of the communication process, teleconsultation services are facilitated. Although MST was used in the context of telemedicine, to the best of our knowledge, it was never used to investigate the complementary or competitive nature of telemedicine tools.

Health care delivery often involves multiple communication tools that must coordinate effectively with each other to achieve the best patient outcomes. MST offers a valuable theoretical lens to address this issue by distinguishing between conveyance and convergence communication processes and aligning them with specific media capabilities. This allows for a more precise evaluation of when tools should substitute for one another versus when they should be integrated to support different stages of communication. Therefore, applying MST enhances our understanding of media choice to explain whether telemedicine tools compete or complement each other.

### Ethical Considerations

This study was a secondary analysis of deidentified data from the NEHRS, conducted by the National Center for Health Statistics (NCHS), US Centers for Disease Control and Prevention (CDC). NEHRS is a national survey of office-based physicians that collects information on the adoption and use of EHRs and related health information technologies. The analytic dataset used in this study was obtained from the public-use NEHRS files, which contain no direct identifiers and adhere to NCHS data confidentiality standards. As this study involved only analysis of existing, fully identified public-use data, no additional institutional review board or human research ethics committee review was sought for this secondary analysis, in accordance with institutional and national guidance on research using public-use survey data. The original NEHRS data collection was conducted by NCHS under applicable federal regulations and ethical guidelines governing human participants research, and participating physicians provided consent at the time of the primary survey. Privacy and confidentiality were maintained throughout the current analysis, and no individual physician, practice, or patient can be identified from the reported results.

## Methods

### Data Resource

This study used data from the NEHRS, a nationally representative survey of office-based ambulatory care physicians in the United States [16-19]. The NEHRS sample is designed to be representative of the broader population of office-based physicians in the United States. It provides crucial insights into EHR use, practice-related details, controlled substance prescribing, health information exchange, telemedicine use, and documentation-related burdens. The survey comprises 32 primary questions, further divided into many subquestions. The initial NEHRS sample in 2021 included 10,302 office-based physicians. After screening for eligibility, 2037 physicians were confirmed to meet the following inclusion criteria: (1) office-based; (2) principally engaged in patient care activities; (3) nonfederally employed; (4) not in the specialties of anesthesiology, pathology, or radiology; and (5) younger than 85 years of age at the time of the survey. Among them, 1875 physicians fully completed the survey by responding to all key items, and these completed responses form the final analytic sample used in this study. All statistical analyses and interpretations in this study are based on the 1875 individual respondents who completed the full survey. The survey provides information not only about physicians’ satisfaction, perceived quality of health care, and the percentage of patient visits conducted through telemedicine, but also includes data on physician gender, age, and clinical setting, among other details [19].

### Dependent Variables and Independent Variables

The dependent variables examined in our study are office-based physicians’ satisfaction with telemedicine technology use (Telemedsat), physicians’ quality-of-health care evaluation in comparison to in-person service (Telemedqual), and percentage of patient visits through telemedicine as a proportion of overall ambulatory care visits. Examining these 3 dependent variables would provide us with a more comprehensive understanding of telemedicine use among ambulatory care physicians. While satisfaction reflects physicians’ personal emotional response to the use of telemedicine tools, physicians’ evaluation of health care quality captures physicians’ retrospective, task-specific judgment about the functional equivalence of telemedicine relative to traditional care delivery [10,47]. The second measure requires physicians to directly compare their own performance, focusing on their perceived ability to maintain clinical standards in a telemedicine setting. The percentage of patient visits via telemedicine tools measures physicians’ active engagement and comfort with telemedicine tools while interacting with patients [11,12,48].

These variables collectively enable us to evaluate the experience, performance, and behavioral adoption of telemedicine tools among ambulatory care physicians. Table 1 presents the descriptive statistics of these 3 dependent variables along with the independent and control variables.

**Table 1:** Descriptive statistics for dependent, independent, and control variables.

Variables and descriptions				Value
**Dependent variables**				
	**Telemedsat^a^, n (%)**			
		Very satisfied	291 (18.05)	
		Somewhat satisfied	671 (41.53)	
		Neither satisfied nor dissatisfied	283 (17.49)	
		Somewhat dissatisfied	261 (16.13)	
		Very dissatisfied	108 (6.80)	
	**Telemedqual^b^, n (%)**			
		Fully	57 (3.52)	
		To a great extent	436 (27.02)	
		To some extent	725 (44.73)	
		To a small extent	337 (20.79)	
		Not at all	62 (3.95)	
	**Telemedpct^c^, n (%)**			
		≥75%	117 (7.77)	
		50%-74%	126 (8.09)	
		25%-49%	421 (27.02)	
		<25%	875 (56.16)	
		None	19 (0.96)	
**Independent variables**				
	**Sum of telemedicine tools used, mean (SD)**			
		SumT^d^	1.97 (0.87)	
	**One telemedicine tool^e^, n (%)**			
		T1^f^ (yes vs no)	1107 (68.08) vs 519 (31.92)	
		T2^g^ (yes vs no)	936 (57.56) vs 690 (42.44)	
		T3^h^ (yes vs no)	707 (43.48) vs 919 (56.52)	
		T4^i^ (yes vs no)	451 (27.74) vs 1175 (72.26)	
	**Bundle telemedicine tools, n (%)**			
		T1T2^j^ vs others	695 (42.74) vs 931 (57.26)	
		T1T3 vs others	469 (28.72) vs 1159 (71.28)	
		T1T4 vs others	330 (20.30) vs 1296 (79.70)	
		T2T3 vs others	318 (19.56) vs 1308 (80.44)	
		T2T4 vs others	219 (13.47) vs 1407 (86.53)	
		T3T4 vs others	136 (8.36) vs 1490 (91.64)	
		T1T2T3 vs others	270 (16.61) vs 1356 (83.39)	
		T1T3T4 vs others	118 (7.26) vs 1508 (92.74)	
		T1T2T4 vs others	187 (11.50) vs 1439 (88.50)	
		T2T3T4 vs others	71 (4.36) vs 1555 (95.64)	
		T1T2T3T4 vs others	65 (4.00) vs 1561 (96.00)	
**Control variables**				
	**Barriers, n (%)**			
		Telemedbarrier1^k^	576 (35.42)	
		Telemedbarrier2^l^	290 (17.83)	
		Telemedbarrier3^m^	423 (26.01)	
		Telemedbarrier4^n^	1078 (66.30)	
		Telemedbarrier5^o^	1152 (70.84)	
	**Facilitator^p^, n (%)**			
		No improvement	757 (46.56)	
		Improvement	869 (53.44)	
	**Physex^q^, n (%)**			
		Male	612 (32.69)	
		Female	1259 (67.31)	
	**Phyage50^r^, n (%)**			
		Age ≤50 years	653 (34.88)	
		Age >50 years	1218 (65.12)	
	**Speccat^s^, n (%)**			
		Primary care specialty	911 (48.69)	
		Surgical specialty	411 (21.97)	
		Medical specialty	549 (29.33)	
	**Numofphy^t^, n (%)**			
		1 physician	432 (23.09)	
		2-3 physicians	367 (19.63)	
		4-10 physicians	557 (28.76)	
		11-50 physicians	292 (15.57)	
		>50 physicians	223 (11.95)	
	**Setting^u^, n (%)**			
		Solo or group practice	1317 (70.35)	
		Other setting	554 (29.65)	

^a^Telemedsat: physicians’ satisfaction (ordinal variable measured on a 5-point Likert scale).

^b^Telemedqual: physicians’ evaluation of health care quality (ordinal variable measured on a 5-point Likert scale).

^c^Telemedpct: percentage of patient visits via telemedicine (ordinal variable measured on a 5-point Likert scale).

^d^SumT: total number of telemedicine tools each physician used (continuous variable).

^e^Distribution of single-tool users: T1 only=121 (7.44%), T2 only=165 (10.15%), T3 only=175 (10.76%), T4 only=77 (4.74%).

^f^T1: telephone audio (binary variable).

^g^T2: videoconference software with audio (eg, Zoom, Ibex, FaceTime; binary variable).

^h^T3: telemedicine platforms not integrated with electronic health records (eg, Doxy. me; binary variable).

^i^T4: telemedicine platforms integrated with electronic health records (eg, updating clinical documentation during a telemedicine visit; binary variable).

^j^T1T2: physicians who use T1 and T2 simultaneously—binary variable (the following variables are expressed in the same way as this).

^k^Telemedbarrier1: limited internet access or speed issues (binary variable).

^l^Telemedbarrier2: telemedicine platform not easy to use or did not meet physician’s needs (binary variable).

^m^Telemedbarrier3: telemedicine is not appropriate for physician’s specialty or type of patients (binary variable).

^n^Telemedbarrier4: limitations in patients’ access to technology (eg, smartphone, computer, tablet, internet; binary variable).

^o^Telemedbarrier5: patients’ difficulty using technology or telemedicine platform (binary variable).

^p^Facilitator: improved reimbursement and relaxation of rules related to use of telemedicine visits (binary variable).

^q^Physex: physicians’ sex (binary variable).

^r^Phyage50: physicians’ age group (binary variable).

^s^Speccat: physicians’ specialty type (multinomial variable).

^t^Numofphy: number of physicians working in the facility (ordinal variable).

^u^Setting: the nature of the practice setting (binary variable).

Independent variables include 4 telemedicine tools: telephone audio (T1); videoconference software with audio (T2; eg, Zoom, Ibex, and FaceTime); telemedicine platforms not integrated with EHR (T3; eg, Doxy.me); and telemedicine platforms integrated with EHR (T4; eg, updating clinical documentation during a telemedicine visit). We counted the total number of telemedicine tools each physician uses and considered them a singular construct (SumT). We also conducted further analysis on different combinations of telemedicine tools (TelemedCombination). For example, physicians used both telephone audio and videoconference software with audio (T1T2), or they simultaneously used telephone audio, videoconference software with audio, and telemedicine platforms not integrated with EHR (eg, Doxy.me; T1T2T3), among other combinations. Notably, in the survey, physicians were asked to indicate which telemedicine tools they used by selecting from a list of 4 options, with no restrictions or dependencies among the tools. This means that each tool could be adopted independently of the others and was not mutually exclusive.

We also consider a set of relevant control variables, including physicians’ sex (Physex), physicians’ age group (Phyage50), physicians’ specialty type (Speccat), number of physicians working in the facility (Numofphy), the nature of the practice (Setting), and perceived barriers and facilitators to telemedicine tools usage. Perceived barriers when using telemedicine tools can be categorized into 5 groups, including limited internet access and speed issues (Telemedbarrier1), platforms that are difficult to use or fail to meet physicians’ needs (Telemedbarrier2), telemedicine may not be suitable for specific specialties or patient types (Telemedbarrier3), limited access to technology for patients (eg, smartphones, computers, tablets, or the internet; Telemedbarrier4), and the difficulties patients experience in navigating telemedicine platforms (Telemedbarrier5). A notable facilitator for telemedicine adoption is the ease of reimbursement for telemedicine visits through third-party providers (Facilitator). To address potential endogeneity concerns, we control physicians’ attitude toward innovation (Telemedbarrier2), patients’ attitude toward telemedicine (Telemedbarrier3 and Telemedbarrier4), policy factors (Facilitator), and organizational factors (Numofphy and Setting).

### Data Analysis

Telemedicine’s impact on physicians’ satisfaction, quality of care, and percentage of patients’ visits are ordinal variables. Thus, we used the ordered logit regression model and the ordered probit regression model (for checking the robustness of our results) to examine the effects of telemedicine on 3 dependent variables, while controlling for other variables that might influence these variables.

y_i_ = β_0_ + β_1_SumT_i_ + γ_1_Telemedbarrier1_i_ γ_2_Telemedbarrier2_i_ + γ_3_Telemedbarrier3_i_ + γ_4_Telemedbarrier4_i_ + γ_5_Telemedbarrier5_i_ + γ_6_Facilitator_i_ + γ_6_Physex_i_ + γ_7_Phyage50_i_ + γ_8_Speccat_i_ + γ_9_Numofphy_i_ + γ_10_Setting_i_ + ε_i_
**(1)**

y_i_ = β_0_ + β_1_T1_i_ + β_2_T2_i_ + β_3_T3_i_ + β_4_T4_i_ + γ_1_Telemedbarrier1_i_ γ_2_Telemedbarrier2_i_ + γ_3_Telemedbarrier3_i_ + γ_4_Telemedbarrier4_i_ + γ_5_Telemedbarrier5_i_ + γ_6_Facilitator_i_ + γ_6_Physex_i_ + γ_7_Phyage50_i_ + γ_8_Speccat_i_ + γ_9_Numofphy_i_ + γ_10_Setting_i_ + ε_i_
**(2)**

y_i_ = β_0_ + β_1_TelemedCombination_i_ + γ_1_Telemedbarrier1_i_ γ_2_Telemedbarrier2_i_ + γ_3_Telemedbarrier3_i_ + γ_4_Telemedbarrier4_i_ + γ_5_Telemedbarrier5_i_ + γ_6_Facilitator_i_ + γ_6_Physex_i_ + γ_7_Phyage50_i_ + γ_8_Speccat_i_ + γ_9_Numofphy_i_ + γ_10_Setting_i_ + ε_i_
**(3)**

For each equation, y_i_ represents the physician’s satisfaction level, physician’s quality valuation of using telemedicine, and the percentage of patients visit via telemedicine, respectively. As for equation 3, TelemedCombination_i_ represents the 11 types of combination of telemedicine tools, including T1T2, T1T3, T1T4, T2T3, T2T4, T3T4, T1T2T3, T1T2T4, T1T3T4, T2T3T4, and T1T2T3T4.

### Correlation Analysis

Phi (φ) correlation coefficients were used to assess the correlations among the binary independent variables (T1, T2, T3, and T4). As shown in Table 2, the correlations were weak, suggesting that the independent variables were free from the concerns of multicollinearity.

**Table 2:** Correlation analysis (phi correlation coefficients and 2-tailed *P* value) among the independent variables. Phi coefficients (φ) were used because both variables were dichotomous (binary).

Variables			T1^a^		T2^b^	T3^c^		T4^d^
**T1**								
	φ	1.00		0.15^e^		–0.03	0.07^e^	
	*P* value	N/A^f^		<.001		.12	<.001	
**T2**								
	φ	0.15^e^		1.00		–0.22^e^	–0.11^e^	
	*P* value	<.001		N/A		<.001	<.001	
**T3**								
	φ	–0.04		–0.22^e^		1.00	–0.17^e^	
	*P* value	.12		<.001		N/A	<.001	
**T4**								
	φ	0.07^e^		–0.11^e^		–0.17^e^	1.00	
	*P* value	<.001		<.001		<.001	N/A	

^a^T1: telephone audio.

^b^T2: videoconference software with audio.

^c^T3: telemedicine platforms not integrated with electronic health records.

^d^T4: telemedicine platforms integrated with electronic health records.

^e^The correlation is significant at a significance level of .01 (2-tailed).

^f^N/A: not applicable.

## Results

### Inferential Statistics

We first used the ordered logit regression model to investigate the effects on each dependent variable (Table 3). We then used an ordered probit regression model to assess the robustness of our estimations (Multimedia Appendix 2).

**Table 3:** Estimated coefficients and SEs for all 3 dependent variables obtained from the ordered logit regression model. Effects of control variables are excluded to conserve space. We used 11 different ordered logit regression models for our 11 different bundles of telemedicine tools: T1T2, T1T3, T1T4, T2T3, T2T4, T3T4, T1T2T3, T1T2T4, T1T3T4, T2T3T4, and T1T2T3T4.

Variables			Physicians’ satisfaction		Physicians’ evaluation of health care quality		Patients’ visit percentage
**SumT^a^**							
	Coefficient (SE)	0.32 (0.06)		0.36 (0.06)		0.26 (0.06)	
	*P* value	<.001		<.001		<.001	
**T1^b^**							
	Coefficient (SE)	0.24 (0.10)		0.36 (0.11)		0.38 (0.12)	
	*P* value	.02		.007		.13	
**T2^c^**							
	Coefficient (SE)	0.29 (0.10)		0.38 (0.10)		0.14 (0.11)	
	*P* value	.004		<.001		.001	
**T3^d^**							
	Coefficient (SE)	0.31 (0.10)		0.27 (0.11)		0.29 (0.11)	
	*P* value	.003		.001		.25	
**T4^e^**							
	Coefficient (SE)	0.52 (0.11)		0.39 (0.11)		0.22 (0.12)	
	*P* value	<.001		.25		.83	
**T1T2^f^**							
	Coefficient (SE)	0.32^g^ (0.10)		0.46^g^ (0.10)		0.31^h^ (0.10)	
	*P* value	.001		<.001		.003	
**T1T3**							
	Coefficient (SE)	0.31^h^ (0.12)		0.29^h^ (0.11)		0.32^h^ (0.11)	
	*P* value	.004		.008		.004	
**T1T4**							
	Coefficient (SE)	0.44^h^ (0.12)		0.31^h^ (0.12)		0.19 (0.13)	
	*P* value	<.001		.01		.13	
**T2T3**							
	Coefficient (SE)	0.27^h^ (0.12)		0.34^h^ (0.12)		0.32^g^ (0.13)	
	*P* value	.02		.006		.01	
**T2T4**							
	Coefficient (SE)	0.57^g^ (0.14)		0.58^g^ (0.14)		0.01 (0.15)	
	*P* value	<.001		<.001		.94	
**T3T4**							
	Coefficient (SE)	0.51^h^ (0.17)		0.31 (0.17)		0.37^g^ (0.18)	
	*P* value	.003		.07		.04	
**T1T2T3**							
	Coefficient (SE)	0.35^g^ (0.13)		0.39^g^ (0.13)		0.41^g^ (0.13)	
	*P* value	.007		.003		.002	
**T1T2T4**							
	Coefficient (SE)	0.60^g^ (0.15)		0.56^g^ (0.15)		0.07 (0.16)	
	*P* value	<.001		<.001		.65	
**T1T3T4**							
	Coefficient (SE)	0.51^h^ (0.18)		0.33^h^ (0.18)		0.45^g^ (0.19)	
	*P* value	.005		.07		.01	
**T2T3T4**							
	Coefficient (SE)	0.59^g^ (0.23)		0.43 (0.23)		0.37 (0.24)	
	*P* value	.01		.06		.12	
**T1T2T3T4**							
	Coefficient (SE)	0.57^g^ (0.24)		0.46 (0.24)		0.48^g^ (0.25)	
	*P* value	.02		.06		.05	
Number of observations^i^			1614		1617		1558

^a^SumT: total number of telemedicine tools each physician used.

^b^T1: telephone audio.

^c^T2: videoconference software with audio (eg, Zoom, Ibex, FaceTime).

^d^T3: telemedicine platforms not integrated with electronic health records (eg, Doxy. me).

^e^T3: telemedicine platforms integrated with electronic health records (eg, updating clinical documentation during a telemedicine visit).

^f^T1T2: physicians who use T1 and T2 simultaneously (the following variables are expressed in the same way as this).

^g^The coefficients for the telemedicine bundles are larger than those for the individual telemedicine tools, and the associations are statistically significant, suggesting that telemedicine tools function complementarily when used as bundles.

^h^The coefficients for the telemedicine bundles are smaller than those for the individual telemedicine tools, and the associations are statistically significant, suggesting that these bundles function as competing rather than complementary tools.

^i^The number of observations varies because each dependent variable, independent variable, and control variable is measured independently and may contain some null or missing values.

We identify the complementary versus competing effects of telemedicine tool bundles based on the relative magnitude of their estimated coefficients in the ordered logit and ordered probit regression models, where the dependent variables are physicians’ satisfaction, physicians’ evaluation of health care quality, and patients’ visit percentage. When the coefficients for the telemedicine bundles exceed those for the individual telemedicine tools, the statistically significant associations suggest that the tools function complementarily when used in combination. For example, regarding the results of the ordered logit regression model, the coefficient of the T1T2 bundle (β=.32; *P*=.001) is higher than that of T1 (β=.24; *P*=.02) and T2 (β=.29; *P*=.004), indicating a complementary effect for this bundle (T1T2) in enhancing physicians’ satisfaction.

Conversely, a bundle is considered competing when its coefficient is significant but lower in value than the coefficients of any of the individual telemedicine tool, suggesting potential competition among tools. For example, the coefficient of the T1T3 bundle (β=.305; *P*=.004) is higher than the coefficient of T1 (β=.24; *P*=.02) but lower than that of T3 (β=.311; *P*=.003), suggesting a competing relationship between these technologies.

We also calculated marginal effects at the mean for each outcome level of the dependent variables to interpret our results meaningfully. Table 4 reports the marginal effects on physicians’ satisfaction levels, Table 5 reports the marginal effects on physicians’ quality-of-health care evaluation, and Table 6 reports the marginal effects on the percentage of patients visiting via telemedicine.

**Table 4:** Marginal effects at the mean and corresponding SEs for physicians’ satisfaction estimated from the ordered logit regression model. Effects of control variables are excluded to conserve space.

Variables		Very dissatisfied	Somewhat dissatisfied	Neither satisfied nor dissatisfied	Somewhat satisfied	Very satisfied
**SumT^a^**						
	Coefficient (SE)	–0.02 (0.00)	–0.03 (0.01)	–0.02 (0.00)	0.02 (0.00)	0.04 (0.01)
	*P* value	<.001	<.001	<.001	<.001	<.001
**T1^b^**						
	Coefficient (SE)	–0.01 (0.01)	–0.02 (0.01)	–0.01 (0.01)	0.02 (0.01)	0.03 (0.01)
	*P* value	.02	.02	.02	.02	.02
**T2^c^**						
	Coefficient (SE)	–0.02 (0.01)	–0.03 (0.01)	–0.01 (0.01)	0.02 (0.01)	0.04 (0.01)
	*P* value	.005	.004	.004	.006	.004
**T3^d^**						
	Coefficient (SE)	–0.02 (0.01)	–0.03 (0.01)	–0.02 (0.01)	0.02 (0.01)	0.04 (0.01)
	*P* value	.003	.003	.003	.004	.003
**T4^e^**						
	Coefficient (SE)	–0.03 (0.01)	–0.05 (0.10)	–0.03 (0.01)	0.03 (0.01)	0.07 (0.02)
	*P* value	<.001	<.001	<.001	<.001	<.001
**T1T2^f^**						
	Coefficient (SE)	–0.02 (0.01)	–0.03 (0.01)	–0.02 (0.01)	0.02 (0.01)	0.04 (0.01)
	*P* value	.001	.001	.001	.002	.001
**T1T3**						
	Coefficient (SE)	–0.02 (0.01)	–0.03 (0.01)	–0.02 (0.01)	0.02 (0.01)	0.04 (0.01)
	*P* value	.005	.004	.005	.006	.004
**T1T4**						
	Coefficient (SE)	–0.025 (0.007)	–0.038 (0.011)	–0.021 (0.006)	0.027 (0.008)	0.058 (0.016)
	*P* value	<.001	<.001	<.001	0.001	<.001
**T2T3**						
	Coefficient (SE)	–0.03 (0.01)	–0.04 (0.01)	–0.02 (0.01)	0.03 (0.01)	0.06 (0.02)
	*P* value	.03	.02	.03	.03	.02
**T2T4**						
	Coefficient (SE)	–0.03 (0.01)	–0.05 (0.01)	–0.03 (0.01)	0.03 (0.01)	0.08 (0.02)
	*P* value	<.001	<.001	<.001	<.001	<.001
**T3T4**						
	Coefficient (SE)	–0.03 (0.01)	–0.05 (0.02)	–0.03 (0.01)	0.03 (0.01)	0.07 (0.02)
	*P* value	.004	.003	.003	.003	.003
**T1T2T3**						
	Coefficient (SE)	–0.02 (0.01)	–0.03 (0.01)	–0.02 (0.01)	0.02 (0.01)	0.05 (0.02)
	*P* value	.009	.008	.008	.009	.007
**T1T2T4**						
	Coefficient (SE)	–0.03 (0.01)	–0.05 (0.01)	–0.03 (0.01)	0.04 (0.01)	0.08 (0.02)
	*P* value	<.001	<.001	<.001	<.001	<.001
**T1T3T4**						
	Coefficient (SE)	–0.03 (0.01)	–0.05 (0.02)	–0.03 (0.01)	0.03 (0.01)	0.07 (0.02)
	*P* value	.006	.006	.005	.007	.005
**T2T3T4**						
	Coefficient (SE)	0.03 (0.01)	–0.05 (0.02)	–0.03 (0.01)	0.04 (0.02)	0.08 (0.03)
	*P* value	.01	.01	.01	.01	.01
**T1T2T3T4**						
	Coefficient (SE)	–0.03 (0.01)	–0.05 (0.02)	–0.03 (0.01)	0.04 (0.02)	0.08 (0.03)
	*P* value	.02	.02	.02	.02	.02

^a^SumT: total number of telemedicine tools each physician used.

^b^T1: telephone audio.

^c^T2: videoconference software with audio (eg, Zoom, Ibex, FaceTime).

^d^T3: telemedicine platforms not integrated with electronic health records (eg, Doxy. me).

^e^T4: telemedicine platforms integrated with electronic health records (eg, updating clinical documentation during a telemedicine visit).

^f^T1T2: physicians who use T1 and T2 simultaneously—binary variable (the following variables are expressed in the same way as this).

**Table 5:** Marginal effects at the mean and corresponding SEs for physicians’ evaluation of health care service quality via telemedicine estimated from the ordered logit regression model. Effects of control variables are excluded to conserve space.

Variables			Not at all		To a small extent		To some extent		To a great extent		Fully	
**SumT^a^**												
	Coefficient (SE)	–0.01 (0.00)		–0.04 (0.01)		–0.01 (0.00)		0.05 (0.01)		0.01 (0.00)		
	*P* value	<.001		<.001		<.001		<.001		<.001		
**T1^b^**												
	Coefficient (SE)	–0.01 (0.00)		–0.04 (0.01)		–0.01 (0.00)		0.05 (0.02)		0.01 (0.00)		
	*P* value	.010		.008		.012		.007		.011		
**T2^c^**												
	Coefficient (SE)	–0.01 (0.00)		–0.04 (0.01)		–0.01 (0.00)		0.06 (0.02)		0.01 (0.00)		
	*P* value	<.001		<.001		.001		<.001		<.001		
**T3^d^**												
	Coefficient (SE)	–0.01 (0.00)		–0.03 (0.01)		–0.01 (0.00)		0.04 (0.02)		0.01 (0.00)		
	*P* value	.001		.001		.002		.001		.002		
**T4^e^**												
	Coefficient (SE)	–0.01 (0.00)		–0.04 (0.01)		–0.01 (0.00)		0.06 (0.02)		0.01 (0.00		
	*P* value	.25		.25		.25		.24		.25		
**T1T2^f^**												
	Coefficient (SE)	–0.02 (0.00)		–0.05 (0.01)		–0.02 (0.00)		0.07 (0.01)		0.02 (0.00)		
	*P* value	<.001		<.001		<.001		<.001		<.001		
**T1T3**												
	Coefficient (SE)	–0.01 (0.00)		–0.03 (0.01)		–0.01 (0.00)		0.04 (0.02)		0.01 (0.00)		
	*P* value	.01		.008		.01		.008		.01		
**T1T4**												
	Coefficient (SE)	–0.01 (0.00)		–0.03 (0.01)		–0.01 (0.01)		0.05 (0.02)		0.01 (0.00)		
	*P* value	.01		.01		.01		.01		.01		
**T2T3**												
	Coefficient (SE)	–0.01 (0.00)		–0.04 (0.01)		–0.01 (0.01)		0.05 (0.02)		0.01 (0.00)		
	*P* value	.009		.007		.01		.006		.01		
**T2T4**												
	Coefficient (SE)	–0.02 (0.01)		–0.06 (0.02)		–0.02 (0.01)		0.09 (0.02)		0.02 (0.01)		
	*P* value	<.001		<.001		<.001		<.001		<.001		
**T3T4**												
	Coefficient (SE)	–0.01 (0.01)		–0.03 (0.02)		–0.01 (0.07)		0.05 (0.03)		0.01 (0.01)		
	*P* value	.08		.07		.08		.07		.08		
**T1T2T3**												
	Coefficient (SE)	–0.01 (0.01)		–0.04 (0.01)		–0.01 (0.01)		0.06 (0.02)		0.01 (0.00)		
	*P* value	.005		.003		.006		.003		.005		
**T1T2T4**												
	Coefficient (SE)	–0.02 (0.01)		–0.06 (0.02)		–0.02 (0.01)		0.08 (0.02)		0.02 (0.01)		
	*P* value	.001		<.001		.001		<.001		.001		
**T1T3T4**												
	Coefficient (SE)	–0.01 (0.01)		–0.04 (0.02)		–0.01 (0.01)		0.04 (0.03)		0.01 (0.01)		
	*P* value	.08		.07		.08		.07		.08		
**T2T3T4**												
	Coefficient (SE)	–0.02 (0.01)		–0.05 (0.03)		–0.02 (0.01)		0.06 (0.03)		0.01 (0.01)		
	*P* value	.07		.06		.07		.06		.07		
**T1T2T3T4**												
	Coefficient (SE)	–0.02 (0.01)		–0.05 (0.03)		–0.02 (0.01)		0.07 (0.04)		0.02 (0.01)		
	*P* value	.06		.06		.06		.06		.06		

^a^SumT: total number of telemedicine tools each physician used.

^b^T1: telephone audio.

^c^T2: videoconference software with audio (eg, Zoom, Ibex, FaceTime).

^d^T3: telemedicine platforms not integrated with electronic health records (eg, Doxy. me).

^e^T4: telemedicine platforms integrated with electronic health records (eg, updating clinical documentation during a telemedicine visit).

^f^T1T2: physicians who use T1 and T2 simultaneously—binary variable (the following variables are expressed in the same way as this).

**Table 6:** Marginal effects at the mean and corresponding SEs for the proportion of patients’ visits conducted via telemedicine estimated from the ordered logit regression model. Effects of control variables are excluded to conserve space.

Variables		None	<25%	25%-49%	50%-74%	≥75%	
**SumT^a^**							
	Coefficient (SE)	–0.00 (0.00)	–0.06 (0.01)	0.03 (0.01)	0.02 (0.00)	0.02 (0.00)	
	*P* value	.002	<.001	<.001	<.001	<.001	
**T1^b^**							
	Coefficient (SE)	–0.01 (0.00)	–0.08 (0.02)	0.04 (0.01)	0.02 (0.01)	0.03 (0.01)	
	*P* value	.16	.13	.13	.13	.14	
**T2^c^**							
	Coefficient (SE)	–0.00 (0.00)	–0.03 (0.02)	0.01 (0.01)	0.01 (0.01)	0.01 (0.01)	
	*P* value	.009	.001	.001	.002	.002	
**T3^d^**							
	Coefficient (SE)	–0.00 (0.00)	–0.06 (0.02)	0.03 (0.01)	0.02 (0.01)	0.02 (0.01)	
	*P* value	.26	.25	.25	.25	.25	
**T4^e^**							
	Coefficient (SE)	–0.00 (0.00)	–0.05 (0.03)	0.02 (0.01)	0.01 (0.01)	0.01 (0.01)	
	*P* value	.83	.83	.83	.83	.83	
**T1T2^f^**							
	Coefficient (SE)	–0.00 (0.00)	–0.06 (0.02)	0.03 (0.01)	0.02 (0.01)	0.02 (0.01)	
	*P* value	.02	.003	.003	.004	.004	
**T1T3**							
	Coefficient (SE)	–0.00 (0.00)	–0.07 (0.02)	0.03 (0.01)	0.02 (0.01)	0.02 (0.01)	
	*P* value	.02	.004	.004	.005	.005	
**T1T4**							
	Coefficient (SE)	–0.00 (0.00)	–0.04 (0.03)	0.02 (0.01)	0.01 (0.01)	0.01 (0.01)	
	*P* value	.15	.13	.13	.13	.13	
**T2T3**							
	Coefficient (SE)	–0.00 (0.00)	–0.07 (0.03)	0.03 (0.01)	0.02 (0.01)	0.02 (0.01)	
	*P* value	.03	.01	.01	.01	.01	
**T2T4**							
	Coefficient (SE)	–0.00 (0.00)	–0.00 (0.03)	0.00 (0.02)	0.00 (0.01)	0.00 (0.01)	
	*P* value	.94	.94	.94	.94	.94	
**T3T4**							
	Coefficient (SE)	–0.00 (0.00)	–0.08 (0.04)	0.04 (0.02)	0.02 (0.01)	0.03 (0.01)	
	*P* value	.06	.04	.04	.04	.04	
**T1T2T3**							
	Coefficient (SE)	–0.00 (0.00)	–0.09 (0.03)	0.04 (0.01)	0.02 (0.01)	0.03 (0.01	
	*P* value	.01	.002	.002	.002	.003	
**T1T2T4**							
	Coefficient (SE)	–0.00 (0.00)	–0.02 (0.03)	–0.02 (0.03)	0.00 (0.01)	0.01 (0.01)	
	*P* value	.66	.65	.65	.65	.65	
**T1T3T4**							
	Coefficient (SE)	–0.01 (0.00)	–0.10 (0.04)	0.04 (0.02)	0.03 (0.01)	0.03 (0.01)	
	*P* value	.03	.01	.02	.02	.02	
**T2T3T4**							
	Coefficient (SE)	–0.01 (0.00)	–0.08 (0.05)	0.04 (0.02)	0.02 (0.01)	0.03 (0.02)	
	*P* value	.14	.12	.12	.12	.12	
**T1T2T3T4**							
	Coefficient (SE)	–0.01 (0.00)	–0.10 (0.05)	0.05 (0.02)	0.03 (0.01)	0.03 (0.02)	
	*P* value	.07	.05	.05	.05	.05	

^a^SumT: total number of telemedicine tools each physician used.

^b^T1: telephone audio.

^c^T2: videoconference software with audio (eg, Zoom, Ibex, and FaceTime).

^d^T3: telemedicine platforms not integrated with electronic health records (eg, Doxy. me).

^e^T4: telemedicine platforms integrated with electronic health records (eg, updating clinical documentation during a telemedicine visit).

^f^T1T2: physicians who use T1 and T2 simultaneously—binary variable (the following variables are expressed in the same way as this).

According to the results in Table 4, parameter estimates for the aggregate model (equation 1) suggest that the summative telemedicine tools have a significant positive relationship with physicians’ satisfaction levels. To be precise, a unit increase in telemedicine tools is associated with approximately 1.8, 2.8, and 1.4 percentage point decreases in the predicted probability of physicians being at the “very dissatisfied,” “somewhat dissatisfied,” and “neither satisfied nor dissatisfied” level of satisfaction. However, a unit increase in telemedicine tools is associated with a 1.9 percentage point increase in the predicted probability of physicians being “somewhat satisfied” and a 4.2 percentage point increase in the predicted probability of physicians being “very satisfied,” respectively.

According to Table 5, the summative telemedicine tools also showed a significant positive association with physicians’ evaluation of telemedicine service quality. The results in Table 5 suggest that a unit increase in telemedicine tools is associated with a decrease of 1.2, 3.9, and 1.2 percentage points in the predicted probability for physicians’ evaluation of telemedicine service quality to be at the level of “not at all,” “to a small extent,” and “some extent.” Contrary to that, a unit increase in telemedicine tools is associated with an increase of 5.2 and 1.2 percentage points in the predicted probability for physicians’ evaluation of health care to be at the level of “to a great extent and fully.”

The results in Table 6 indicate that a unit increase in telemedicine tools is associated with a decrease of 0.3 and 5.5 percentage points in the predicted probability that the percentage of patients visiting via telemedicine will be at the level of “none” or “<25%.” In contrast, a unit increase in telemedicine tools is associated with increases of 2.5, 1.5, and 1.8 percentage points in the predicted probability for this dependent variable to be “25%-49%,” “50%-74%,” and “≥75%,” respectively.

Parameter estimates for the disaggregate model (equation 2) suggest that each individual telemedicine tool has a significant positive relationship with physicians’ satisfaction level and physicians’ quality of health care evaluation (Tables 4 and 5). However, while T1 and T3 showed a significant positive association with the percentage of patients visiting via telemedicine, T2 and T4 showed no such relationship (Table 6).

Regarding the parameter estimates for all the different telemedicine tools combination models (equation 3), we found that each combination of telemedicine tools shares a significant positive association with the physician’s satisfaction level (Table 4). However, the combinations of T3T4, T1T3T4, and T2T3T4 do not share any significant association with physicians’ quality-of-health care evaluation (Table 5). Additionally, the combination of T1T4, T2T4, T1T2T4, and T2T3T4 also does not share any significant association with the percentage of patients’ visits via telemedicine (Table 6).

### Additional Tests

We also considered the possibility of a moderating effect of patient visits via telemedicine tools, given the time period during which the data were collected (the pandemic era). This situation may have been caused by patients’ limited access to in-person care rather than the bundle of technologies that the physician was using. To test this possibility, we conducted an additional analysis. According to Table 7, the results indicate that the moderator yielded no statistically significant interaction effects.

**Table 7:** Additional tests for examining the moderating effect of patient visits performed via telemedicine based on the ordered logit and ordered probit regression models. Effects of control variables are excluded to conserve space.

Variables			Physicians’ satisfaction				Physicians’ evaluation of health care quality		
**SumT^a^ × Telemedpct^b^**									
	Coefficient (SE)	–0.03 (0.06)		–0.02 (0.04)	–0.06 (0.06)			–0.03 (0.03)	
	*P* value	.68		.64	.36			.38	
**T1^c^× Telemedpct**									
	Coefficient (SE)	–0.06 (0.12)		–0.03 (0.07)	–0.01 (0.12)			–0.00 (0.07)	
	*P* value	.995		.97	.39			.47	
**T2^d^× Telemedpct**									
	Coefficient (SE)	–0.15 (0.11)		–0.08 (0.06)	0.00 (0.11)			–0.00 (0.06)	
	*P* value	.31		.25	.72			.66	
**T3^e^× Telemedpct**									
	Coefficient (SE)	0.06 (0.11)		0.01 (0.06)	–0.06 (0.11)			–0.03 (0.06)	
	*P* value	.65		.77	.90			.80	
**T4^f^× Telemedpct**									
	Coefficient (SE)	0.08 (0.12)		0.06 (0.07)	–0.12 (0.12)			–0.05 (0.07)	
	*P* value	.69		.77	.37			.38	
**T1T2^g^× Telemedpct**									
	Coefficient (SE)	–0.10 (0.11)		–0.05 (0.06)	–0.02 (0.11)			–0.02 (0.06)	
	*P* value	.36		.45	.84			.77	
**T1T3 × Telemedpct**									
	Coefficient (SE)	–0.05 (0.11)		–0.04 (0.07)	–0.07 (0.11)			–0.04 (0.07)	
	*P* value	.65		.52	.52			.59	
**T1T4 × Telemedpct**									
	Coefficient (SE)	–0.01 (0.13)		0.01 (0.08)	–0.12 (0.13)			–0.05 (0.08)	
	*P* value	.92		.93	.38			.51	
**T2T3 × Telemedpct**									
	Coefficient (SE)	–0.01 (0.12)		–0.02 (0.07)	–0.00 (0.12)			–0.01 (0.07)	
	*P* value	.96		.75	.98			.94	
**T2T4 × Telemedpct**									
	Coefficient (SE)	0.05 (0.16)		0.04 (0.09)	–0.07 (0.16)			–0.02 (0.09)	
	*P* value	.75		.66	.64			.80	
**T3T4 × Telemedpct**									
	Coefficient (SE)	0.22 (0.18)		0.13 (0.10)	–0.10 (0.17)			–0.05 (0.10)	
	*P* value	.23		.21	.50			.63	
**T1T2T3 × Telemedpct**									
	Coefficient (SE)	0.00 (0.13)		–0.01 (0.08)	0.01 (0.13)			0.00 (0.08)	
	*P* value	.99		.91	.96			.99	
**T1T2T4 × Telemedpct**									
	Coefficient (SE)	0.03 (0.17)		0.03 (0.10)	–0.11 (0.16)			–0.05 (0.09)	
	*P* value	.88		.78	.50			.62	
**T1T3T4 × Telemedpct**									
	Coefficient (SE)	0.03 (0.19)		0.01 (0.11)	–0.13 (0.19)			–0.07 (0.11)	
	*P* value	.88		.90	.49			.53	
**T2T3T4 × Telemedpct**									
	Coefficient (SE)	0.08 (0.08)		0.21 (0.15)	–0.04 (0.23)			–0.03 (0.13)	
	*P* value	.17		.16	.85			.85	
**T1T2T3T4 × Telemedpct**									
	Coefficient (SE)	0.36 (0.26)		0.21 (0.15)	–0.08 (0.24)			–0.04 (0.14)	
	*P* value	.16		.16	.74			.75	
Number of observations^h^			1548	1548		1551			1551

^a^SumT: total number of telemedicine tools each physician used.

^b^Telemedpct: percentage of patient visits via telemedicine.

^c^T1: telephone audio.

^d^T2: videoconference software with audio (eg, Zoom, Ibex, and FaceTime).

^e^T3: telemedicine platforms not integrated with electronic health records (eg, Doxy. me).

^f^T4: telemedicine platforms integrated with electronic health records (eg, updating clinical documentation during a telemedicine visit).

^g^T1T2: physicians who use T1 and T2 simultaneously (the following variables are expressed in the same way as this).

^h^The number of observations varies because each dependent variable, independent variable, and control variable is measured independently and may contain some null or missing values.

## Discussion

### Principal Results

Our study addresses the complementary versus competing nature of telemedicine tools. Our empirical analysis reveals that although both individual and combined uses of telemedicine tools positively affect physicians’ satisfaction, quality of health care evaluation, and the percentage of patients’ visits via telemedicine, various telemedicine tools compete rather than complement each other. Our study suggests that the relationships among telemedicine tools are not always positive; instead, their combined use can produce either complementary or competing effects, depending on how their functionalities interact within the context of physicians’ clinical workflows.

### Interpretation of Findings

For physicians, we found that various combinations of telemedicine tools significantly influence their overall satisfaction and partially affect their evaluation of health care quality. These findings are consistent with prior studies reporting generally high physician satisfaction and perceived usefulness of telemedicine [49,50], as well as comparable quality of care between telemedicine and in-person consultations [51]. However, these earlier studies did not examine how different combinations of telemedicine tools shape physicians’ satisfaction and quality-of-care assessments. Our study extends this understanding by highlighting how the complementary use of telemedicine tools can influence these outcomes. Our findings suggest that satisfaction and the perceived quality of care are influenced by the coherence of the technology stack, with videoconferencing software (T2) acting as a powerful core. Bundles that include T2 create a complementary effect by providing a rich, high-fidelity communication channel essential for clinical assessment. The ability to observe visual cues and nonverbal information is fundamental to accurate diagnosis and building effective communication between physicians and patients. Notably, the combination of T2 with an integrated EHR (T4) represents the ideal modern workflow, creating an efficient interaction environment where the physician can focus on the patient through a high-quality video interface while the EHR seamlessly handles documentation. Even a simpler T2 and T1 (telephone) bundle is complementary, as the telephone serves as a reliable backup that reduces physician worry about technical failures and connectivity disruptions.

Conversely, bundles lacking video-based technological support generate a competing effect, characterized by cognitive overload and workflow fragmentation. Combinations such as T1 with platform T3 or T4 force physicians to split their attention between a low-fidelity audio call and a separate, complex software interface. Furthermore, the combination of 2 different platforms (T3 + T4) exemplifies competing technologies and cognitive overload, leading to technological redundancy, confusion, and a fragmented patient management process that detracts from the physician’s core responsibilities [52-54].

From the perspective of MST, these findings can be interpreted in terms of differences in media capabilities and process alignment. Videoconferencing (T2) provides high levels of immediacy of feedback, variety of symbols, and transmission velocity, enabling physicians to convey and interpret complex medical information effectively. When such rich media are integrated with complementary systems (like EHRs), they enhance conveyance and convergence processes. Thus, both information exchange and shared understanding result in higher satisfaction and perceived quality. In contrast, bundles that rely on low-synchronicity tools (eg, telephone or nonintegrated platforms) fail to support these communication processes, increasing the cognitive effort required to maintain clinical coherence and reducing the perceived effectiveness of telemedicine-oriented patient visits.

Additionally, the combination of T3 (nonintegrated telemedicine platforms) and T4 (EHR-integrated telemedicine platforms) exhibits a competing effect on physicians’ satisfaction and does not significantly improve care quality, which may be due to their underlying system architecture. T3 platforms, such as Doxy.me, operate independently from the EHR system and function primarily as stand-alone communication tools. In contrast, T4 tools are embedded within the EHR environment, supporting real-time documentation and patient data access during telemedicine consultations. When these tools are used in combination, as in T3T4 or T1T3T4, physicians are required to switch between 2 unconnected noncompatible systems, which disrupts workflow and necessitates repeated actions such as reentering patient information or clinical notes [52-54]. This lack of interoperability increases the administrative and information searching burden and undermines efficiency.

In contrast to physicians’ satisfaction and quality evaluation outcomes, physicians report a higher percentage of patient visits via telemedicine when the technological configuration is broader and more inclusive, incorporating multiple platforms (eg, T2T3, T3T4, T1T2T3T4). These complementary effects arise because more comprehensive bundles enable physicians to engage in a broader range of patient types and clinical scenarios through various modalities. For example, combining video (T2) and telemedicine platforms (T3 or T4) allows clinicians to conduct both structured and flexible consultations, manage follow-ups, and accommodate patients with differing technical capacities or conditions. From a physician’s standpoint, such diversity enhances operational flexibility and reduces barriers to providing telemedicine services, thus naturally increasing the proportion of visits conducted remotely.

However, simpler 2-tool bundles (eg, T1T2 and T1T3) show a competing effect on the percentage of patient visits via telemedicine. There might be 2 reasons leading to this competing effect. From the physicians’ perspective, while these configurations are easy to operate, they lack the integration required to sustain a consistent telemedicine workflow across different patient groups. A telephone-plus-video, or telephone-plus-stand-alone-platform setup without an integrated EHR may be functional for ad hoc consultations but insufficient for record-keeping or health care information exchange. From the patients’ perspective, they may not perceive much difference between an in-person visit and interacting with physicians only via telephone or videoconference. Consequently, many patients may still prefer face-to-face consultations, as they feel more direct and personal.

Interestingly, combinations that include EHR-integrated tools (T4), such as T1T4, T2T4, T1T2T4, or T2T3T4, do not significantly impact the percentage of patient visits via telemedicine. We believe there are 2 main reasons for this outcome. First, physicians must complete patients’ electronic records while simultaneously communicating with patients via video or phone, which makes each consultation more time-consuming [55]. As a result, the total number of telemedicine visits that can be completed in a day decreases. Second, the log-in and verification procedures add complexity to the communication process between physicians and patients, leading physicians to prefer phone consultations (audio call or FaceTime call) for many simple cases. Consequently, the combination of T4 with other tools shows no statistically significant impact on the overall percentage of telemedicine visits.

We also conducted an additional test to investigate the potential moderating role of patient visits via telemedicine tools, as data collected during the pandemic period may reflect patients’ limited choices rather than the effect of physicians’ technology bundles. However, our results do not support this moderation effect. We believe there are 2 main reasons for this. First, based on prior studies (eg, Moulaei et al [48], Reed et al [40], and Tzeng et al. [56]), patient visits via telemedicine tools are consistently conceptualized as an outcome shaped by demographic, contextual, and technological factors. Second, theoretically, drawing on MST [43], we believe that patient telehealth usage reflects media-task fit and can be better understood as a behavioral outcome. To ensure theoretical and empirical coherence, we therefore treat the percentage of patient visits via telemedicine tools as one of our dependent variables.

### Limitations and Future Directions

One limitation of this study is the use of a self-reported measure to capture physicians’ perceived quality of care, which introduces potential bias due to self-reporting. Although the survey item aimed to minimize this bias by prompting a direct comparison between telemedicine visits and in-person visits, the subjective nature of the measure limits the objectivity of our findings. Future research should apply objective clinical performance indicators or third-party assessments to validate physicians’ evaluations.

Another limitation of this study is that the telemedicine tool usage variable does not capture the frequency or intensity of use for each tool. Thus, our study cannot determine whether physicians’ satisfaction primarily comes from one tool or from a combination of tools. Our analysis, therefore, treats tool combinations as reported usage bundles. Future research should incorporate more data on usage frequency, duration, or physician preference to capture the priority of individual telemedicine tools. This would enable researchers to investigate the effects of specific technologies within multitool combinations and gain a deeper understanding of how different tools contribute to physicians’ satisfaction.

Due to data limitations, our study cannot thoroughly test the possibility of endogenous variables. Although we have attempted to identify any possible source of missing variable bias theoretically, we have not been successful in doing so. Future research should investigate such possibilities by identifying a suitable instrumental variable. Our empirical analysis used data from the national survey conducted by the NCHS at the CDC [16-19], which notably excludes several specialties, specifically radiology, pathology, and anesthesiology. Consequently, the applicability of our findings to these clinical contexts is limited. This exclusion stems from the inherently procedural and in-person nature of these fields (eg, anesthesia administration and diagnostic imaging), which may restrict the deployment of current telemedicine tools. We consider this data limitation as one of the key limitations of our research. Future research should incorporate these specialties to fully assess the generalizability of telemedicine usage across the broader clinical spectrum.

Last, this study focuses exclusively on the perspectives of ambulatory care physicians, which limits the ability to assess how tool combinations affect patient-centered outcomes or patient satisfaction. Future research should collect patient-centered data to build a more comprehensive understanding of the effectiveness and acceptance of telemedicine tools from the patient’s perspective.

### Conclusions

Our study contributes to practice that enhances understanding of how telemedicine tools can be effectively used in health care, particularly in postpandemic scenarios. Each tool offers unique advantages, so using the right tool for the right situation is not just a choice, but a crucial decision that can significantly improve both physician satisfaction and patient engagement in telemedicine. This insight provides clear recommendations to providers and platform developers, emphasizing the key to the success of telemedicine implementation—creating a seamless experience for physicians. Such a system needs to be designed in a manner that ensures the complementary behavior of different tools based on physicians’ specific demands, highlighting the complexity of various clinical and communicative tasks.

## Appendix

Multimedia Appendix 1 Synthesis of literature review.

Multimedia Appendix 2 Conducting robustness tests for all three dependent variables using the ordered probit regression model.

## Abbreviations

CDC: US Centers for Disease Control and Prevention
EHR: electronic health record
MST: media synchronicity theory
NCHS: National Center for Health Statistics
NEHRS: 2021 National Electronic Health Records Survey
Numofphy: number of physicians working in the facility
Phyage50: physicians’ age group
Physex: physicians’ sex
Setting: the nature of the practice
Speccat: physicians’ specialty type
SumT: total number of telemedicine tools each physician used
T1: telephone audio
T2: videoconference software with audio
T3: telemedicine platforms not integrated with electronic health records
T4: telemedicine platforms integrated with electronic health records
Telemedbarrier1: limited internet access or speed issues
Telemedbarrier2: telemedicine platform not easy to use or did not meet physician’s needs
Telemedbarrier3: telemedicine is not appropriate for physician’s specialty or type of patients
Telemedbarrier4: limitations in patients’ access to technology
Telemedbarrier5: patients’ difficulty using technology or telemedicine platform
Telemedpct: percentage of patient visits conducted through telemedicine as a proportion of overall ambulatory care visits
Telemedqual: physicians’ quality-of-health care evaluation in comparison to in-person service
Telemedsat: office-based physicians’ satisfaction with telemedicine technology use
