# Supplementary material for: Competition or Complementarity Among Telemedicine Tools in Ambulatory Care Practice: Cross-Sectional Analysis
Source: JMIR Med Inform. 2025 Dec 23;13:e75246. doi: 10.2196/75246 (PMC12775760; doi:10.2196/75246)
Supplement: Multimedia Appendix 1 [file medinform_v13i1e75246_app1.docx]

**Table S1.** Synthesis of literature review.

|  |  | **Technology** | | | | | **Clinic focus** | | **Outcomes** | | | | | | | | | | |
| --- | --- | --- | --- | --- | --- | --- | --- | --- | --- | --- | --- | --- | --- | --- | --- | --- | --- | --- | --- |
|  |  | Telemedicine Tools | Communication Channels | | | | Clinical Settings | Targeted Populations | Usability and Accessibility | Accuracy and Quality | Rehabilitation and Recovery | Medication Adherence | Safety Standards | Cost-Effective Solutions | Digital Literacy | Privacy Protection | Telemedicine Capacity | Motivation and Well-Bing | Efficiency |
| Reference | Citation |  | Audio | Video | Text | Others |  |  |  |  |  |  |  |  |  |  |  |  |  |
| 23 | op den Akker et al. 2015 | Real-time physical activity coaching system |  |  |  | √ | Physiology | Old patients with chronic diseases |  |  | √ |  |  |  |  |  |  | √ |  |
| 24 | Shanthanna et al. 2020 | Audio-video platforms | √ | √ |  |  | Primary care settings | Patients with chronic diseases |  | √ |  |  |  |  |  |  | √ |  |  |
| 25 | Shah & Badawy, 2021 | Audio-video platforms | √ | √ |  |  | Pediatrics | Female patients |  |  | √ |  |  | √ |  |  |  |  |  |
| 26 | Boehm et al. 2020 | Videoconference | √ | √ |  |  | Urology | Male patients with urological diseases |  |  | √ |  |  |  |  |  |  |  |  |
| 27 | Gagnon et al. 2014 | Electronic health record (EHR) |  |  | √ |  | General settings | Physicians |  | √ |  |  |  |  | √ | √ | √ | √ | √ |
| 28 | Khera et al. 2020 | Videoconference | √ | √ |  |  | Cardiology | Patients with cardiovascular disease |  |  | √ | √ |  |  |  |  |  |  |  |
| 29 | Sengupta et al. 2024 | Multiple telemedicine tools | √ | √ | √ | √ | Office-based clinical settings | General patients |  |  |  |  |  | √ |  |  | √ |  | √ |
| 30 | Zhang & Saltman, 2022 | EHR |  |  | √ |  | General clinical settings | General patients |  | √ |  |  |  |  |  |  |  |  |  |
| 33 | Ratwani et al. 2019 | EHR | √ | √ | √ |  | Emergency department | General patients | √ |  |  |  | √ |  |  |  |  |  | √ |
| 35 | Atherton et al. 2012 | Email |  |  | √ |  | General clinical settings | General patients |  | √ |  |  |  |  |  |  |  |  | √ |
| 36 | Donaghy et al. 2019 | Video consultation |  | √ |  |  | Office-based clinical settings | Working people and people with mobility or mental health problems | √ |  |  |  |  |  | √ |  |  | √ | √ |
| 37 | Everson & Butler, 2020 | EHR |  |  | √ |  | Nonfederal and acute care hospitals | US patients | √ |  |  |  |  |  |  |  |  |  |  |
| 38 | Bates et al. 2014 | EHR |  |  | √ |  | General US clinical settings | High-risk, high-cost clinically complex patients |  |  |  |  |  | √ |  | √ | √ |  |  |
| 39 | Hayes 2019 | EHR and Computerized Physician Order Entry (CPOE) |  |  | √ |  | General US clinical settings | General patients |  |  |  |  |  |  |  |  |  | √ |  |
| 40 | Asgari et al. 2024 | EHR |  |  | √ |  | Ambulatory care | General patients |  |  |  |  |  |  |  |  |  | √ |  |
| 41 | Campbell et al. 2023 | Virtual Consultations |  | √ |  |  | General clinical settings | Patients with mental issues |  | √ |  |  |  |  | √ |  |  |  | √ |
| 42 | Car et al. 2020 | Video consultations |  | √ |  |  | Primary and specialist care | UK patients | √ |  |  |  |  |  |  |  |  |  | √ |
| 43 | Reed et al. 2020 | Multiple telemedicine tools | √ | √ | √ | √ | Primary care | General patients | √ |  |  |  |  | √ |  |  |  |  |  |
| 44 | Seuren et al. 2020 | Video consultations |  | √ |  |  | Cardiology | Patients with heart failure | √ |  |  |  |  | √ |  |  |  |  | √ |
| 45 | Shao et al. 2023 | Multiple telemedicine tools | √ | √ | √ | √ | Ambulatory care | Patients with chronic diseases |  | √ |  |  |  | √ |  |  |  |  |  |
| 47 | Tan & Yan, 2020 | Mobile-phone-based APPs | √ | √ | √ | √ | Ambulatory care | General patients |  | √ |  |  |  |  |  |  |  | √ |  |
| 48 | Yan et al. 2016 | Teleconsultation | √ | √ |  |  | Chinese private and public hospital | General patients | √ |  |  |  |  |  |  |  |  |  |  |
| 49 | Damico et al. 2022 | Multiple telemedicine tools | √ | √ | √ | √ | Radiation oncology | Patients with cancers |  | √ |  |  |  | √ |  |  |  |  |  |
| 52 | Holmgren et al. 2024 | EHR |  |  | √ |  | Ambulatory care | General patients | √ |  |  |  |  |  |  |  |  | √ |  |
| 54 | Ratwani et al. 2018 | EHR |  |  | √ |  | Pediatrics | Female patients | √ |  |  |  | √ |  |  |  |  |  |  |
| 55 | Tzeng et al. 2022 | Outpatient virtual clinics platforms | √ | √ | √ | √ | Taiwan large general hospital | General patients | √ |  |  |  |  | √ |  |  |  |  |  |
