# Supplementary material for: Competition or Complementarity Among Telemedicine Tools in Ambulatory Care Practice: Cross-Sectional Analysis
Source: JMIR Med Inform. 2025 Dec 23;13:e75246. doi: 10.2196/75246 (PMC12775760; doi:10.2196/75246)
Supplement: Multimedia Appendix 2 [file medinform_v13i1e75246_app2.docx]

**Appendix B. Robustness Tests**

**Table B1.** Estimated coefficients and standard errors from robustness checks using the ordered probit regression model.^a^

| Variables | | Physicians’ satisfaction | Physicians’ evaluation of healthcare quality | Patients’ visit percentage |
| --- | --- | --- | --- | --- |
| SumT^b^ | | | | |
|  | Coefficient (Std. Error) | 0.19 (0.03) | 0.21 (0.03) | 0.16 (0.04) |
|  | *P* value | <.001 | <.001 | <.001 |
| T1^c^ | | | | |
|  | Coefficient (Std. Error) | 0.15 (0.06) | 0.22 (0.06) | 0.22 (0.07) |
|  | *P* value | .001 | .006 | .20 |
| T2^d^ | | | | |
|  | Coefficient (Std. Error) | 0.17 (0.06) | 0.21 (0.06) | 0.10 (0.06) |
|  | *P* value | .002 | <.001 | .001 |
| T3^e^ | | | | |
|  | Coefficient (Std. Error) | 0.19 (0.06) | 0.16 (0.06) | 0.19 (0.06) |
|  | *P* value | .01 | <.001 | .34 |
| T4^f^ | | | | |
|  | Coefficient (Std. Error) | 0.31 (0.07) | 0.25 (0.07) | 0.11 (0.07) |
|  | *P* value | .80 | .26 | .65 |
| T1T2^g^ | | | | |
|  | Coefficient (Std. Error) | 0.19^i^ (0.06) | 0.26^i^ (0.06) | 0.20^j^ (0.06) |
|  | *P* value | .001 | <.001 | .001 |
| T1T3 | | | | |
|  | Coefficient (Std. Error) | 0.17^j^ (0.06) | 0.18^j^ (0.06) | 0.19^j^ (0.07) |
|  | *P* value | .005 | .004 | .004 |
| T1T4 | | | | |
|  | Coefficient (Std. Error) | 0.27^j^ (0.07) | 0.20^j^ (0.07) | 0.10 (0.07) |
|  | *P* value | <.001 | .005 | .18 |
| T2T3 | | | | |
|  | Coefficient (Std. Error) | 0.15^j^ (0.07) | 0.20^j^ (0.07) | 0.21^i^ (0.08) |
|  | *P* value | .03 | .005 | .005 |
| T2T4 | | | | |
|  | Coefficient (Std. Error) | 0.35^i^ (0.08) | 0.34^i^ (0.08) | 0.01 (0.09) |
|  | *P* value | <.001 | <.001 | .90 |
| T3T4 | | | | |
|  | Coefficient (Std. Error) | 0.30^j^ (0.10) | 0.20^j^ (0.10) | 0.22 (0.10) |
|  | *P* value | .003 | .04 | .04 |
| T1T2T3 | | | | |
|  | Coefficient (Std. Error) | 0.20^i^ (0.08) | 0.23^i^ (0.08) | 0.26^i^ (0.08) |
|  | *P* value | .009 | .002 | .001 |
| T1T2T4 | | | | |
|  | Coefficient (Std. Error) | 0.37^i^ (0.09) | 0.33^i^ (0.09) | 0.05 (0.09) |
|  | *P* value | <.001 | <.001 | .60 |
| T1T3T4 | | | | |
|  | Coefficient (Std. Error) | 0.31^i^ (0.11) | 0.22^j^ (0.11) | 0.26^i^ (0.11) |
|  | *P* value | .004 | .04 | .02 |
| T2T3T4 | | | | |
|  | Coefficient (Std. Error) | 0.37^i^ (0.14) | 0.28^i^ (0.13) | 0.24 (0.14) |
|  | *P* value | .007 | .04 | .09 |
| T1T2T3T4 | | | | |
|  | Coefficient (Std. Error) | 0.36^i^ (0.14) | 0.30^i^ (0.14) | 0.30^i^ (0.15) |
|  | *P* value | .01 | .04 | .04 |
| Number of observations^h^ | | 1,614 | 1,617 | 1,558 |
| ^a^Effects of control variables are excluded to conserve space.  ^b^SumT: Total number of telemedicine tools each physician used.  ^c^T1: Telephone audio.  ^d^T2: Videoconference software with audio (e.g., Zoom, Ibex, FaceTime).  ^e^T3: Telemedicine platforms not integrated with EHR (e.g., Doxy. me).  ^f^T4: Telemedicine platforms integrated with EHR (e.g., updating clinical documentation during a telemedicine visit).  ^g^T1T2: Physicians who use T1 and T2 simultaneously: Binary variable (the following variables are expressed in the same way as this).  ^h^The number of observations varies because each dependent variable, independent variable, and control variable is measured independently and may contain some null or missing values.  ^i^The coefficients for the telemedicine bundles are larger than those for the individual telemedicine tools, and the associations are statistically significant, suggesting that telemedicine tools function complementarily when used as bundles.  ^j^The coefficients for the telemedicine bundles are smaller than those for the individual telemedicine tools, and the associations are statistically significant, suggesting that these bundles function as competing rather than complementary tools. | | | | |

**Table B2.** Estimated marginal effects at the mean and standard errors for physicians’ satisfaction levels based on the ordered probit regression model.^a^

| Variables | | Very dissatisfied | Somewhat dissatisfied | Neither satisfied nor dissatisfied | Somewhat satisfied | Very satisfied |
| --- | --- | --- | --- | --- | --- | --- |
| SumT^b^ | | | | | | |
|  | Coefficient (S.E.) | -0.02 (0.00) | -0.03 (0.01) | -0.01 (0.00) | 0.02 (0.00) | 0.04 (0.01) |
|  | *P* value | <.001 | <.001 | <.001 | <.001 | <.001 |
| T1^c^ | | | | | | |
|  | Coefficient (S.E.) | -0.02 (0.01) | -0.02 (0.01) | -0.01 (0.01) | 0.01 (0.01) | 0.04 (0.01) |
|  | *P* value | .001 | .001 | .001 | .001 | .001 |
| T2^d^ | | | | | | |
|  | Coefficient (S.E.) | -0.02 (0.01) | -0.02 (0.01) | -0.01 (0.00) | 0.02 (0.01) | 0.04 (0.01) |
|  | *P* value | .002 | .002 | .002 | .002 | .002 |
| T3^e^ | | | | | | |
|  | Coefficient (S.E.) | -0.02 (0.01) | -0.03 (0.01) | -0.01 (0.00) | 0.02 (0.01) | 0.04 (0.01) |
|  | *P* value | .01 | .01 | .01 | .02 | .01 |
| T4^f^ | | | | | | |
|  | Coefficient (S.E.) | -0.03 (0.01) | -0.05 (0.01) | -0.02 (0.01) | 0.03 (0.01) | 0.07 (0.02) |
|  | *P* value | .80 | .80 | .80 | .80 | .80 |
| T1T2^g^ | | | | | | |
|  | Coefficient (S.E.) | -0.02 (0.01) | -0.03 (0.01) | -0.01 (0.00) | 0.02 (0.01) | 0.04 (0.01) |
|  | *P* value | .001 | .001 | .001 | .001 | .001 |
| T1T3 | | | | | | |
|  | Coefficient (S.E.) | -0.02 (0.01) | -0.03 (0.01) | -0.01 (0.05) | 0.02 (0.01) | 0.04 (0.01) |
|  | *P* value | .005 | .005 | .005 | .006 | .005 |
| T1T4 | | | | | | |
|  | Coefficient (S.E.) | -0.03 (0.01) | -0.04 (0.01) | -0.02 (0.01) | 0.03 (0.01) | 0.06 (0.02) |
|  | *P* value | <.001 | <.001 | <.001 | <.001 | <.001 |
| T2T3 | | | | | | |
|  | Coefficient (S.E.) | -0.02 (0.01) | -0.02 (0.01) | -0.01 (0.01) | 0.01 (0.01) | 0.04 (0.02) |
|  | *P* value | .03 | .03 | .03 | .03 | .03 |
| T2T4 | | | | | | |
|  | Coefficient (S.E.) | -0.04 (0.01) | -0.05 (0.01) | -0.03 (0.01) | 0.03 (0.01) | 0.08 (0.02) |
|  | *P* value | <.001 | <.001 | <.001 | <.001 | <.001 |
| T3T4 | | | | | | |
|  | Coefficient (S.E.) | -0.03 (0.01) | -0.04 (0.02) | -0.02 (0.01) | 0.03 (0.01) | 0.07 (0.02) |
|  | *P* value | .004 | .003 | .003 | .004 | .003 |
| T1T2T3 | | | | | | |
|  | Coefficient (S.E.) | -0.02 (0.01) | -0.03 (0.01) | -0.01 (0.01) | 0.02 (0.01) | 0.05 (0.02) |
|  | *P* value | .01 | .009 | .009 | .01 | .009 |
| T1T2T4 | | | | | | |
|  | Coefficient (S.E.) | -0.04 (0.01) | -0.05 (0.01) | -0.03 (0.01) | 0.04 (0.01) | 0.09 (0.02) |
|  | *P* value | <.001 | <.001 | <.001 | <.001 | <.001 |
| T1T3T4 | | | | | | |
|  | Coefficient (S.E.) | -0.03 (0.01) | -0.05 (0.02) | -0.02 (0.01) | 0.03 (0.01) | 0.07 (0.03) |
|  | *P* value | .004 | .004 | .004 | .005 | .004 |
| T2T3T4 | | | | | | |
|  | Coefficient (S.E.) | -0.04 (0.02) | -0.05 (0.02) | -0.03 (0.01) | 0.04 (0.01) | 0.09 (0.03) |
|  | *P* value | .008 | .007 | .007 | .009 | .007 |
| T1T2T3T4 | | | | | | |
|  | Coefficient (S.E.) | -0.04 (0.02) | -0.05 (0.02) | -0.03 (0.01) | 0.03 (0.01) | 0.08 (0.03) |
|  | *P* value | .01 | .01 | .01 | .01 | .01 |
| ^a^Effects of control variables are excluded to conserve space.  ^b^SumT: Total number of telemedicine tools each physician used.  ^c^T1: Telephone audio.  ^d^T2: Videoconference software with audio (e.g., Zoom, Ibex, FaceTime).  ^e^T3: Telemedicine platforms not integrated with EHR (e.g., Doxy. me).  ^f^T4: Telemedicine platforms integrated with EHR (e.g., updating clinical documentation during a telemedicine visit).  ^g^T1T2: Physicians who use T1 and T2 simultaneously: Binary variable (the following variables are expressed in the same way as this). | | | | | | |

**Table B3.** Estimated marginal effects at the mean and standard errors for physicians’ evaluation of telemedicine healthcare service quality based on the ordered probit regression model.^a^

| Variables | | Not at all | To a small extent | To some extent | To a great extent | Fully |
| --- | --- | --- | --- | --- | --- | --- |
| SumT^b^ | | | | | | |
|  | Coefficient (S.E.) | -0.01 (0.00) | -0.04 (0.01) | -0.01 (0.00) | 0.05 (0.01) | 0.02 (0.00) |
|  | *P* value | <.001 | <.001 | <.001 | <.001 | <.001 |
| T1^c^ | | | | | | |
|  | Coefficient (S.E.) | -0.02 (0.00) | -0.04 (0.01) | -0.01 (0.00) | 0.05 (0.01) | 0.02 (0.01) |
|  | *P* value | .007 | .006 | .012 | .006 | .008 |
| T2^d^ | | | | | | |
|  | Coefficient (S.E.) | -0.01 (0.00) | -0.04 (0.01) | -0.01 (0.00) | 0.05 (0.01) | 0.02 (0.00) |
|  | *P* value | <.001 | <.001 | .001 | <.001 | <.001 |
| T3^e^ | | | | | | |
|  | Coefficient (S.E.) | -0.01 (0.00) | -0.03 (0.01) | -0.01 (0.00) | 0.04 (0.01) | 0.01 (0.00) |
|  | *P* value | .001 | <.001 | .002 | <.001 | .001 |
| T4^f^ | | | | | | |
|  | Coefficient (S.E.) | -0.02 (0.01) | -0.04 (0.01) | -0.01 (0.00) | 0.06 (0.02) | 0.02 (0.01) |
|  | *P* value | .26 | .26 | .27 | .26 | .26 |
| T1T2^g^ | | | | | | |
|  | Coefficient (S.E.) | -0.02 (0.00) | -0.05 (0.01) | -0.01 (0.00) | 0.06 (0.01) | 0.02 (0.00) |
|  | *P* value | <.001 | <.001 | <.001 | <.001 | <.001 |
| T1T3 | | | | | | |
|  | Coefficient (S.E.) | -0.01 (0.00) | -0.03 (0.01) | -0.01 (0.00) | 0.04 (0.01) | 0.01 (0.01) |
|  | *P* value | .005 | .004 | .009 | .004 | .005 |
| T1T4 | | | | | | |
|  | Coefficient (S.E.) | -0.01 (0.01) | -0.04 (0.01) | -0.01 (0.00) | 0.05 (0.02) | 0.01 (0.01) |
|  | *P* value | .006 | .005 | .010 | .004 | .007 |
| T2T3 | | | | | | |
|  | Coefficient (S.E.) | -0.01 (0.01) | -0.04 (0.01) | -0.01 (0.00) | 0.05 (0.02) | 0.01 (0.01) |
|  | *P* value | .006 | .005 | .010 | .005 | .007 |
| T2T4 | | | | | | |
|  | Coefficient (S.E.) | -0.02 (0.01) | -0.06 (0.02) | -0.02 (0.01) | 0.08 (0.02) | 0.02 (0.01) |
|  | *P* value | <.001 | <.001 | <.001 | <.001 | <.001 |
| T3T4 | | | | | | |
|  | Coefficient (S.E.) | -0.01 (0.01) | -0.04 (0.02) | -0.01 (0.01) | 0.05 (0.02) | 0.01 (0.01) |
|  | *P* value | .05 | .05 | .06 | .05 | .05 |
| T1T2T3 | | | | | | |
|  | Coefficient (S.E.) | -0.02 (0.01) | -0.04 (0.01) | -0.01 (0.00) | 0.05 (0.02) | 0.02 (0.01) |
|  | *P* value | .003 | .002 | .005 | .002 | .003 |
| T1T2T4 | | | | | | |
|  | Coefficient (S.E.) | -0.02 (0.01) | -0.06 (0.02) | -0.02 (0.01) | 0.07 (0.02) | 0.02 (0.01) |
|  | *P* value | <.001 | <.001 | <.001 | <.001 | <.001 |
| T1T3T4 | | | | | | |
|  | Coefficient (S.E.) | -0.02 (0.01) | -0.04 (0.02) | -0.01 (0.01) | 0.05 (0.03) | 0.02 (0.01) |
|  | *P* value | .04 | .04 | .04 | .04 | .04 |
| T2T3T4 | | | | | | |
|  | Coefficient (S.E.) | -0.02 (0.01) | -0.05 (0.03) | -0.01 (0.01) | 0.07 (0.03) | 0.02 (0.01) |
|  | *P* value | .04 | .04 | .04 | .04 | .04 |
| T1T2T3T4 | | | | | | |
|  | Coefficient (S.E.) | -0.02 (0.01) | -0.06 (0.03) | -0.01 (0.01) | 0.07 (0.03) | 0.02 (0.01) |
|  | *P* value | .04 | .04 | .04 | .04 | .04 |
| ^a^Effects of control variables are excluded to conserve space.  ^b^SumT: Total number of telemedicine tools each physician used.  ^c^T1: Telephone audio.  ^d^T2: Videoconference software with audio (e.g., Zoom, Ibex, FaceTime).  ^e^T3: Telemedicine platforms not integrated with EHR (e.g., Doxy. me).  ^f^T4: Telemedicine platforms integrated with EHR (e.g., updating clinical documentation during a telemedicine visit).  ^g^T1T2: Physicians who use T1 and T2 simultaneously: Binary variable (the following variables are expressed in the same way as this). | | | | | | |

**Table B4.** Estimated marginal effects at mean and standard errors for patients’ visit via telemedicine based on the ordered probit regression model.^a^

| Variables | | None | Less than 25% | 25% to 49% | 50% to 74% | 75% or more |
| --- | --- | --- | --- | --- | --- | --- |
| SumT^b^ | | | | | | |
|  | Coefficient (S.E.) | -0.01 (0.00) | -0.05 (0.01) | 0.02 (0.01) | 0.02 (0.00) | 0.02 (0.01) |
|  | *P* value | <.001 | <.001 | <.001 | <.001 | <.001 |
| T1^c^ | | | | | | |
|  | Coefficient (S.E.) | -0.01 (0.00) | -0.07 (0.02) | 0.03 (0.01) | 0.02 (0.01) | 0.03 (0.01) |
|  | *P* value | .21 | .19 | .19 | .19 | .19 |
| T2^d^ | | | | | | |
|  | Coefficient (S.E.) | -0.00 (0.00) | -0.03 (0.02) | 0.01 (0.01) | 0.01 (0.01) | 0.01 (0.01) |
|  | *P* value | .005 | .001 | .001 | .001 | .001 |
| T3^e^ | | | | | | |
|  | Coefficient (S.E.) | -0.01 (0.00) | -0.06 (0.02) | 0.03 (0.01) | 0.02 (0.01) | 0.03 (0.01) |
|  | *P* value | .35 | .35 | .35 | .35 | .35 |
| T4^f^ | | | | | | |
|  | Coefficient (S.E.) | -0.00 (0.00) | -0.04 (0.02) | 0.02 (0.01) | 0.01 (0.01) | 0.02 (0.01) |
|  | *P* value | .65 | .65 | .65 | .65 | .65 |
| T1T2^g^ | | | | | | |
|  | Coefficient (S.E.) | -0.01 (0.00) | -0.07 (0.02) | 0.03 (0.01) | 0.02 (0.01) | 0.03 (0.01) |
|  | *P* value | .006 | .001 | .001 | .002 | .002 |
| T1T3 | | | | | | |
|  | Coefficient (S.E.) | -0.01 (0.00) | -0.06 (0.02) | 0.03 (0.01) | 0.02 (0.01) | 0.03 (0.01) |
|  | *P* value | .01 | .004 | .004 | .004 | .004 |
| T1T4 | | | | | | |
|  | Coefficient (S.E.) | -0.00 (0.00) | -0.03 (0.03) | 0.01 (0.01) | 0.01 (0.01) | 0.01 (0.01) |
|  | *P* value | .20 | .18 | .18 | .18 | .18 |
| T2T3 | | | | | | |
|  | Coefficient (S.E.) | -0.01 (0.00) | -0.07 (0.03) | 0.03 (0.01) | 0.02 (0.01) | 0.03 (0.01) |
|  | *P* value | 0.02 | .005 | .005 | .006 | .006 |
| T2T4 | | | | | | |
|  | Coefficient (S.E.) | -0.00 (0.00) | -0.00 (0.03) | 0.00 (0.01) | 0.00 (0.01) | 0.00 (0.01) |
|  | *P* value | .90 | .90 | .90 | .90 | .90 |
| T3T4 | | | | | | |
|  | Coefficient (S.E.) | -0.01 (0.00) | -0.07 (0.04) | 0.03 (0.02) | 0.02 (0.01) | 0.03 (0.01) |
|  | *P* value | .05 | .04 | .04 | .04 | .04 |
| T1T2T3 | | | | | | |
|  | Coefficient (S.E.) | -0.01 (0.00) | -0.09 (0.03) | 0.04 (0.01) | 0.03 (0.01) | 0.03 (0.01) |
|  | *P* value | .006 | .001 | .001 | .001 | .001 |
| T1T2T4 | | | | | | |
|  | Coefficient (S.E.) | -0.00 (0.00) | -0.02 (0.03) | 0.01 (0.01) | 0.00 (0.01) | 0.01 (0.01) |
|  | *P* value | .60 | .60 | .60 | .60 | .60 |
| T1T3T4 | | | | | | |
|  | Coefficient (S.E.) | -0.01 (0.00) | -0.09 (0.04) | 0.04 (0.02) | 0.02 (0.01) | 0.03 (0.01) |
|  | *P* value | .03 | .02 | .02 | .02 | .02 |
| T2T3T4 | | | | | | |
|  | Coefficient (S.E.) | -0.01 (0.00) | -0.08 (0.05) | 0.03 (0.02) | 0.02 (0.01) | 0.03 (0.02) |
|  | *P* value | .11 | .09 | .10 | .10 | .10 |
| T1T2T3T4 | | | | | | |
|  | Coefficient (S.E.) | -0.01 (0.01) | -0.10 (0.05) | 0.04 (0.02) | 0.03 (0.01) | 0.04 (0.02) |
|  | *P* value | .06 | .04 | .04 | .04 | .04 |
| ^a^Effects of control variables are excluded to conserve space.  ^b^SumT: Total number of telemedicine tools each physician used.  ^c^T1: Telephone audio.  ^d^T2: Videoconference software with audio (e.g., Zoom, Ibex, FaceTime).  ^e^T3: Telemedicine platforms not integrated with EHR (e.g., Doxy. me).  ^f^T4: Telemedicine platforms integrated with EHR (e.g., updating clinical documentation during a telemedicine visit).  ^g^T1T2: Physicians who use T1 and T2 simultaneously: Binary variable (the following variables are expressed in the same way as this). | | | | | | |
